# Forebrain NgR1 Overexpression Impairs DA Release Suggesting Synergy of Local and Global Synaptic Plasticity Mechanisms

**DOI:** 10.3389/fnsyn.2020.545854

**Published:** 2020-12-10

**Authors:** Emma Arvidsson, Sarolta Gabulya, Alvin Tore Brodin, Tobias Erik Karlsson, Lars Olson

**Affiliations:** Department of Neuroscience, Karolinska Institutet, Stockholm, Sweden

**Keywords:** Nogo receptor 1, Nogo, plasticity, substantia nigra, striatum, MemoFlex mouse, dopamine release, *in vivo* chronoamperometry

## Abstract

Structural synaptic reorganizations needed to permanently embed novel memories in the brain involve complex plasticity-enhancing and plasticity-inhibiting systems. Increased neural activity is linked to rapid downregulation of Nogo receptor 1 (NgR1), needed to allow local structural synaptic plasticity. This local regulation of plasticity is thought to be moderated by global systems, such as the ascending cholinergic and monoaminergic systems, adding significance to locally increased neural activity. Here we address the reverse possibility that the global systems may also be influenced by the status of local plasticity. Using NgR1-overexpressing mice, with impaired plasticity and long-term memory, we measured the ability to release dopamine (DA), implicated in regulating plasticity and memory. *In vivo* chronoamperometric recording with high temporal and spatial resolution revealed severe impairment of potassium chloride (KCl)-induced increase of extracellular DA in the dorsal striatum of mice overexpressing NgR1 in forebrain neurons. A similar, but lesser, impairment of DA release was seen following amphetamine delivery. In contrast, potassium chloride-evoked DA release in NgR1 knockout (KO) mice led to increased levels of extracellular DA. That NgR1 can impair DA signaling, thereby further dampening synaptic plasticity, suggests a new role for NgR1 signaling, acting in synergy with DA signaling to control synaptic plasticity.

**Significance Statement:**The inverse correlation between local NgR1 levels and magnitude of KCl-inducible amounts of DA release in the striatum reinforces the rule of NgR1 as a regulator of structural synaptic plasticity and suggests synergy between local and global plasticity regulating systems.

## Introduction

While lasting memories are the result of a large number of interacting mechanisms, there is strong evidence that the final executive representation of a lasting memory is an altered structure of the neuronal network. *In vivo* imaging of postsynaptic sites in rodents suggests that individual newly formed synapses can exist for years, as required if the altered synaptic pattern is to carry lasting memories (Yang et al., 2009). Using the same technique, it has been found that rapid eye movement (REM) sleep promotes pruning of newly formed spines and that this process is critical for the ability to form new and stable connections (Li et al., 2017). Hence, memory formation is a delicate process where spines are created or removed, strengthened or weakened depending on need. The structural changes needed to form a specific memory occur across large parts of the cortex. These can all be reached at the same time by the ascending modulatory systems from the brain stem. A complex and precisely organized molecular machinery consisting of plasticity-enhancing and plasticity-inhibiting systems is necessary to implement the required structural reorganizations that permanently embed novel memories in the brain. Nogo receptor 1 (NgR1) is a key inhibitory regulator that is expressed in forebrain neurons and mediates nerve growth inhibition in response to Nogo and other ligands (Schwab, 2010). Neuronal activity downregulates neuronal NgR1 (Josephson et al., 2003; Nordgren et al., 2013; Karlsson et al., 2017) and hence increases local plasticity, including dendritic complexity (Karlsson et al., 2016), while the inability to downregulate NgR1 impairs long-term memory (Karlén et al., 2009).

The global regulatory systems consist of ascending cholinergic and monoaminergic projections innervating the forebrain. Here, we focus on the dopamine (DA) system, originating at subcortical levels, providing modulatory messages to higher-level circuitry and strengthening specific cellular contacts based on ongoing events. It has been shown that the DA input impacts structural synaptic plasticity in the motor cortex (Guo et al., 2015) and striatum (Fasano et al., 2013). Here, we address the reverse possibility that the global DA input might be influenced by the status of local plasticity as controlled by NgR1. Using different protocols targeting DA release and kinetics monitored by *in vivo* chronoamperometry, we find that potassium chloride (KCl)- and amphetamine-induced increase of extracellular DA levels in the dorsal striatum is markedly less in NgR1-overexpressing mice and that KCl-induced release of DA is instead increased in mice lacking NgR1. This suggests that the presumed plasticity-enhancing effects of the DA circuitry is enhanced by NgR1 downregulation.

## Materials and Methods

### Animals

All animal procedures were performed in accordance with the animal care committee’s regulations. Experiments took place during the light phase, between 6:30 AM and 6:30 PM. Food and water were provided *ad libitum*. Adult (8–11 weeks of age) mice of both sexes were used. Control mice were cage mates and thus age-matched per experiment. NgR1-overexpressing mice (MemoFlex) have previously been characterized (Karlén et al., 2009; Karlsson et al., 2016). Like MemoFlex mice, NgR1 knockout (KO) mice do not show any obvious health problems.

### Electrode Preparation and Calibration

**Figure 1:**
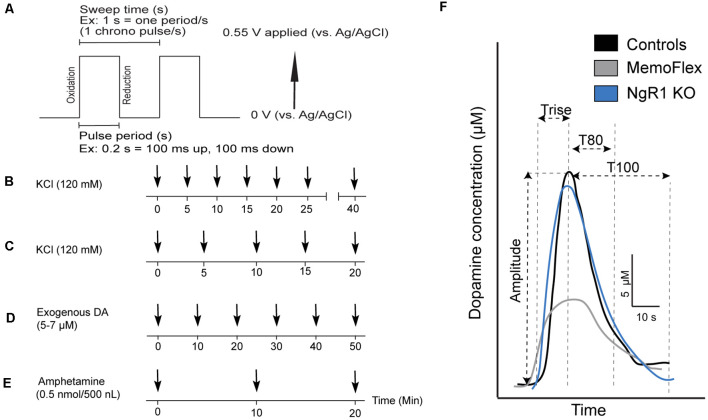
High-speed *in vivo* chronoamperometry recording setup and dopamine (DA) kinetic parameters. **(A)** Recordings were performed by applying a square-wave pulse sufficient to oxidize DA at the coated working electrode. **(B–E)** Stimulation protocols. **(B)** DA release was evoked in MemoFlex mice by six consecutive 100-nl potassium chloride (KCl; 120 mM) pressure ejections applied locally and spaced by 5 min, followed by one final KCl ejection after 15 min of rest in MemoFlex (*n* = 7) and control (*n* = 7) mice. **(C)** Protocol for KCl delivery and DA recording from Nogo receptor 1 (NgR1) knockout (KO) mice. **(D)** Protocol for local delivery of exogenous DA analyzing reuptake. **(E)** Protocol for local delivery of amphetamine and recording of DA levels. As 120 mM KCl also stimulates the release of other neurotransmitters such as glutamate, 500 nl of 0.5 nmol amphetamine was chosen as a more selective DA stimulant. **(F)** Representative traces of DA release kinetics following the first KCl stimulation in MemoFlex, NgR1 KO, and control mice. *Amplitude*, peak DA concentration (μM) from baseline; *Trise*, time (seconds) between injection and maximum peak concentration; *T80*, time (seconds) from maximum peak concentration until an 80% decrease of the maximum amplitude; *T100* (now shown), time (seconds) from maximum amplitude until 100% decrease from maximum peak concentration, used as a measure of DA clearance.

For electrochemical experiments, single carbon fiber electrodes (30-μm outer diameter, 100–200-μm length; Quanteon) were used. Prior to implantation, electrodes were coated at 200°C with three to four layers of a 5% Nafion solution (Sigma–Aldrich) to prevent interference from anionic compounds such as ascorbic acid. DA recordings were achieved using Fast Analytical Sensing Technology (FAST; Quanteon) according to methodological principles described previously (Gerhardt and Burmeister, 2000; Figure 1A). Electrodes were tested for sensitivity to ascorbic acid (250 μm) and calibrated with three accumulating concentrations of DA (Sigma–Aldrich; 2–6 μm) *in vitro*. Electrodes displaying selectivity ratios exceeding 500:1 over ascorbic acid and linear response to DA (*r*^2^ > 0.995) were used for *in vivo* experiments.

### *In vivo* Dopamine Recordings

Recordings were collected from the dorsal striatum of urethane-anesthetized (12.5%) Memoflex, NgR1 KO, and littermate controls. Animals were positioned in a stereotactic frame and maintained at a body temperature of 38°C. Local analgesic was applied subcutaneously on the head. A small incision was then made over the scalp, a cotton swab was used to dry and expose the skull bone before drilling. A microdrill with a diamond burr (diameter 1.4 mm) was used to enable implantation; the dura was removed by using a small needle (G27). Prior to implantation, the bregma (where the coronal suture is intersected perpendicularly by the sagittal suture) and lambda (the point of meeting of the sagittal and the lambdoid sutures) were aligned dorsoventrally. Stereotaxic coordinates according to a standard brain atlas (Franklin and Paxinos, 2013) were 1.1 mm in front of and ±1.5 mm lateral to the bregma. Electrodes were lowered to a position 3.2 mm below the dura mater. Electrode placement in the dorsal striatum was verified by postmortem dissection in all samples. An Ag/AgCl reference electrode was placed in the cerebral cortex contralateral to, and distant from, the recording site. In the first experiment, DA release was evoked by six consecutive pressure ejections of 100–120 nl of 120 mM KCl, applied locally at 5-min intervals and, for the final seventh ejection, 15 min after the sixth injection. Basal release capacity was defined by stimulation 1; recovery capacity was defined as a significant increase in DA amplitude upon stimulation 7 compared to stimulation 6. Regain of basal release capability was defined as the absence of a significant difference in DA amplitude between stimulation 1 and stimulation 7 (Arvidsson et al., 2014). Total experimental time was 70–90 min (Memoflex: *n* = 8, three females, five males; controls: *n* = 8, six females, two males). In the next set of experiments, five consecutive pressure ejections of 100–120 nl of 120 mM KCl (Sigma–Aldrich), applied locally at 5-min intervals, were performed, comparing controls and NgR1 KO mice (NgR1 KO: *n* = 4 males; controls: *n* = 4 males). In a following experiment, six consecutive pressure ejections of exogenous DA (5–7 μ M; Sigma–Aldrich) were applied locally to analyze dopamine transporter (DAT) function (Cass et al., 1993; MemoFlex: *n* = 7, four females, three males; controls: *n* = 7, four females, three males). In the final experiments, three local pressure ejections of amphetamine (0.5 nmol/500 nl; Sigma–Aldrich) were used to evoke DA release in the dorsal striatum (MemoFlex: *n* = 8, four females, four males; controls: *n* = 8, five females, three males). Recordings were performed during applications of repeated square-wave pulse (+0.55 V for 100 ms with 0.0 V 100 ms resting intervals, with respect to a reference electrode) repeated at a frequency of 5 Hz using the FAST (FAST-16 Quantion system). An average signal was automatically calculated for every five cycles by the recording software, resulting in the temporal resolution of 1 data point per second (1 Hz).

### Levels of Nogo Receptor 1, Dopamine Transporter, Tyrosine Hydroxylase, D1, and D2 in the Striatum

Using Western blots, the protein levels of NgR1 (INQ0216121, 1:2,500), tyrosine hydroxylase (TH; ab112, 1:5,000), D1 (ab81296, 1:2,500), D2 (ab85367, 1:10,000), and DAT (ab128848, 1:2,500) were examined and analyzed using ImageJ. SYPRO Ruby staining was used to adjust for loading and transfer differences. Cryostat 20-μm sections of formalin-perfused brain tissue were immunolabeled for TH+ fiber density (ab 112, 1:1,600) in striatum and co-labeled for NgR1 (INQ0216121, 1:400) to directly compare locations of NgR1 and TH. Images were taken with a Zeiss 800-airy microscope using optimal settings for the airy detector.

### Data Analysis

Reduction/oxidation (redox) ratios were calculated at the peak of every response to confirm the identity of the analyte contributing to the electrochemical signal. DA typically displays a redox ratio of 0.7–0.9 *in vivo*, whereas possible interfering electrochemical species have lower redox ratios [e.g., 0 for ascorbic acid and 0.1 for 5-hydroxytryptamine (5-HT); Gerhardt and Hoffman, 2001; Supplementary Figure 1]. To ensure that DA contributed to the electrochemical signal, only experiments displaying redox ratios exceeding 0.7 were included in the data analysis. The average redox ratio for the experiments presented here was 0.73. The effects of genotype on KCl-evoked DA release were statistically analyzed using a linear mixed model (R, Lme4 package) followed by *post hoc* testing using estimated marginal means (emmeans) and the multivariate T (MVT) distribution to adjust for multiple testing. In cases where only two groups were compared, a *t*-test was instead performed. In all experiments where genders were mixed, we first used a model that included gender. If no significant gender effect was found (true in all experiments), the gender variable was dropped from the model. Four parameters were derived from the electrochemical signal: (1) amplitude, defined as the peak DA concentration (micromoles) from baseline; (2) *t*-rise, time (seconds) between ejection and maximum peak concentration; (3) *t*-80, the time (seconds) from maximum peak concentration until 80% decrease of the maximum amplitude, as a measure of DA clearance; and (4) T100, the time (seconds) from peak maximum until all extracellular DA surrounding the electrode tip is removed (Figure 1F; Cass et al., 1993; Hebert et al., 1996; Hoffman et al., 1998).

## Results

### Impaired Potassium Chloride-Induced Increase of Extracellular Dopamine in the Dorsal Striatum of Nogo Receptor 1-Overexpressing Mice

**Figure 2:**
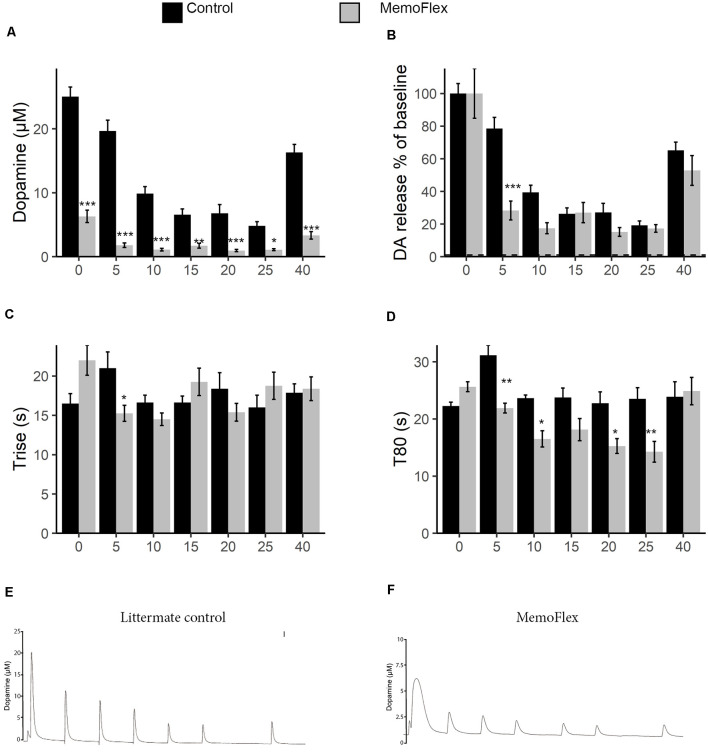
Potassium chloride (KCl)-induced release and clearance of extracellular dopamine (DA) in the dorsal striatum of MemoFlex and control mice. **(A)** Quantification of DA amplitude in response to six repeated KCl stimuli and, after a 15-min pause, of partial recovery of release amplitude in response to a seventh stimulation in control (black) and MemoFlex (gray) mice. **(B)** The same data as in a but normalized to baseline release per genotype. **(C,D)** Trise and T80 DA release parameters in control and MemoFlex mice. Mean values obtained for each stimulation (1–6) within each group. *p < 0.05, **p < 0.01, as determined using estimated marginal means (emmeans) with multivariate T (MVT) adjustment, expressed as mean ± SEM. MemoFlex: *n* = 8, three females, five males; controls: *n* = 8, six females, two males. **(E,F)** Example curves of DA release in control **(E)** and MemoFlex **(F)** mice. ***p < 0.001.

To assess the ability to release DA, six stimulations with KCl were applied with 5-min intervals followed by a 15-min resting period, after which a final stimulation was given (protocol Figure 1B). This protocol was implemented to allow the analysis of basal release capacity (stimulation 1), ability to maintain release during repeated stimuli (stimulations 2–6), and ability to recover release properties after a longer break (stimulation 7). When the system was naive, control mice released >5 times the amount of DA released by NgR1-overexpressing mice (*p* < 0.0001; Figures 2A,E,F). During stimulations 1–5, there were also markedly lower levels of KCl-induced extracellular DA levels in MemoFlex compared to control mice (Genotype *p* < 2.2 * 10^−16^, Treatment *p* < 2.2 * 10^−16^, Genotype × Treatment *p* < 2.2 * 10^−16^). Control mice recovered partially after 15 min of rest, as seen by comparing the sixth and seventh stimulations (*p* < 0.0001). However, the control mice still had lower extracellular levels of DA following 15 min of recovery compared to the first KCl treatment (seventh vs. first stimulation: *p* < 0.0001). In MemoFlex striatum, there was no significant increase of KCl-induced DA release after 15 min of rest (seventh vs. sixth stimulation: *p* = 0.7276; Figure 2A). As there was such a big difference in DA release, we also quantified the release normalized to the amount of DA released at the first stimulation per group (Figure 2B). Analyzing data this way we found that the biggest difference in change of release compared to baseline occured at the first repeated stimulation (5 min; *p* < 0.001). We then analyzed how long it took to release DA. We found a significant interaction when analyzing the time it took to release DA (Trise) during stimulations 1–5 (Genotype *p* = 0.96, Time *p* = 0.06, Genotype × Time *p* < 6.2 * 10^−6^; Figure 2C). This was mostly due to changes in how rapid DA was released during the first two time points. During the later stages of the experiment, Trise was similar between the groups. When we analyzed how long it took to reduce the DA concentration to 80% of the pre-KCl levels (T80), we found a clear difference (Genotype *p* = 6.06 * 10^−6^, Time *p* = 2.4 * 10^−7^, Genotype * Time *p* = 4.3 * 10^−5^; Figure 2D). Both groups started quite similar, but with time, MemoFlex mice cleared DA more and more rapidly.

### Clearance of Exogenous Dopamine in the Striatum Is Not Influenced by Nogo Receptor 1 Overexpression

**Figure 3:**
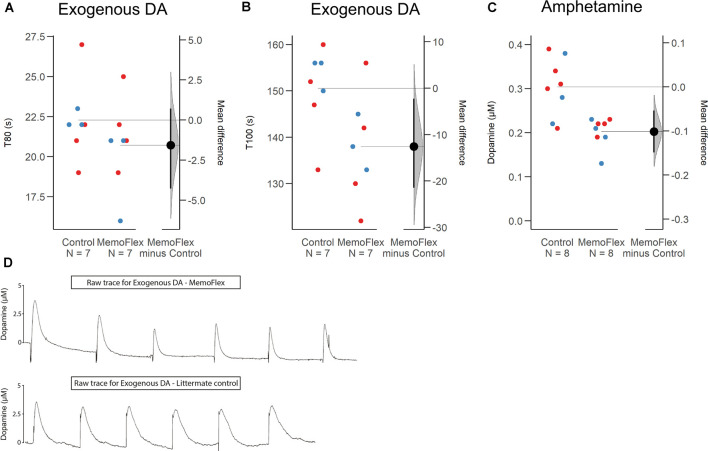
Clearance of exogenous dopamine (DA) and amphetamine-induced release of DA in the dorsal striatum of MemoFlex mice and littermate controls. **(A,B)** Mean values of T80 and T100 obtained from six consecutive ejections of exogenous DA. Male: blue; Female: red. **(C)** Mean values of DA released by three amphetamine ejections. **(D)** Raw traces of exogenous DA in MemoFlex (top) and control mice (bottom). *p < 0.05, **p < 0.01, as detected by two-tailed Student’s *t*-test, expressed as mean ± SEM. MemoFlex: *n* = 7, four females, three males; controls: *n* = 7, four females, three males.

To obtain information about whether KCl-induced increase of extracellular DA was impaired due to impaired release or impaired reuptake and/or metabolism, we delivered small amounts of exogenous DA to the dorsal striatum (protocol Figure 1C). Analyzing the mean of six consecutive deposits of exogenous DA applied at a constant amplitude, there was no difference in T80 between the two groups (*p* = 0.28; Figure 3D) slightly shorter (8.5%) in MemoFlex mice compared to controls (*p* = 0.04) presumably because controls release a larger amount of endogenous DA than MemoFlex mice (Figure 3B).

### Amphetamine-Induced Dopamine Release in the Dorsal Striatum Is Impaired in Nogo Receptor 1-Overexpressing Mice

In addition to KCl, we also tested possible effects of local amphetamine, a potent and more selective evoker of DA release (protocol Figure 1D). We found that the mean extracellular DA levels evoked by three consecutive amphetamine ejections applied 10 min apart was significantly lower in MemoFlex mice than in control mice (*p* = 0.0018; Figure 3C).

### Nogo Receptor 1, Tyrosine Hydroxylase, Dopamine Transporter, D1, and D2 Receptors in the Dorsal Striatum of Nogo Receptor 1-Overexpressing Mice

**Figure 4:**
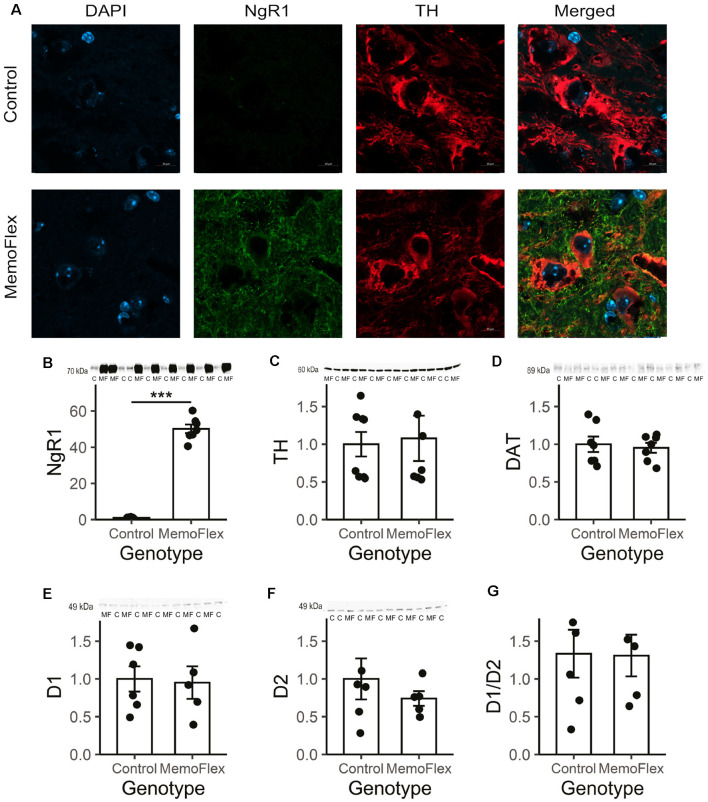
Tyrosine hydroxylase (TH), Nogo receptor 1 (NgR1), and dopamine (DA) receptor D2 in the dorsal striatum of NgR1-overexpressing mice. **(A)** Immunolabeling with 4′,6-diamidino-2-phenylindole (DAPI; blue), TH (green), and NgR1 (red) of the substantia nigra pars compacta of control and NgR1-overexpressing MemoFlex mice. The TH-positive neurons are equally strong, but the amount of NgR1-immunoreactive neurons surrounding them differs. **(B)** NgR1 expression in the striatum. **(C)** TH expression did not differ between controls and MemoFlex. **(D)** Dopamine transporter (DAT) levels were not affected by overexpression. **(E)** Western blot shows a >50-fold increase of NgR1 protein in the MemoFlex striatum. Western blot did not reveal any significant alteration of the levels of D1 **(E)** or D2 **(F)** receptor protein or the ratio of D1/D2 **(G)** in the MemoFlex striatum. ***p < 0.001.

To further investigate the considerable difference in DA release between MemoFlex and control mice, we looked for possible differences in the dopaminergic system (Figures 4A–G). Immunohistochemistry demonstrated that the fluorescence intensity and morphology of TH+ neurons in substantia nigra pars compacta in MemoFlex and control mice were similar. As expected, there was a strong overexpression of NgR1-like immunoreactivity in fibers surrounding the TH+ cells in MemoFlex mice, but we did not detect measurable levels of NgR1 inside the TH+ cells (Figure 4A). Western blots confirmed the histochemical findings of increased NgR1 expression by showing a >50-fold increase of NgR1 protein in the MemoFlex striatum (Figure 4B). Supporting the lack of changes of the dopaminergic innervation of the striatum in MemoFlex mice, Western blots showed similar amounts of TH protein in MemoFlex and control mice (Figure 4C). This is consistent with previous findings that striatal DA levels are not affected in MemoFlex mice (Karlén et al., 2009). Interestingly, TH levels in our experiments appeared to be separated into low and high expressors, divided equally between the groups. Measurements of DAT did not reveal any difference between control and MemoFlex mice (Figure 4D). Likewise, levels of D1 and D2 DA receptor proteins were not significantly altered in the striatum of MemoFlex mice, as compared to control mice (Figures 4E,F). We also assessed if the balance between D1 and D2 signaling could have been altered by assessing the ratio of D1 to D2 receptors. In line with the data of the absolute receptor levels, the ratio was not significantly altered (Figure 4G).

### Potassium Chloride-Induced Increase of Extracellular Dopamine in the Dorsal Striatum of Nogo Receptor 1 Knockout Mice

Since overexpression of NgR1 inhibited the local release of DA, we next asked if absence of NgR1 might instead increase DA release (protocol Figure 1E). Results show a significant increase of KCl-induced levels of extracellular DA in the dorsal striatum of NgR1 KO mice compared to controls (*p* = 0.04; Supplementary Figure 1).

## Discussion

Densely packed brain stem and midbrain monoamine cell groups (Dahlström and Fuxe, 1964) with ascending axons innervate vast forebrain areas. This creates global ascending pathways such as the mesocorticolimbic pathway and the nigrostriatal pathway. These projections provide information about saliency and motivational value of an external stimulus and are commonly referred to as the brain reward system (Ikemoto and Bonci, 2014). Local input to these cell groups can thus exert widespread “global” forebrain effects. At the cellular level, DA induces the expression of learning-related genes *via* the transcription factor c-Fos (Wang and McGinty, 1997), and at the level of synapses, DA has been shown to affect many aspects of plasticity, including long-term potentiation (LTP; Hosp and Luft, 2013) and modulating the threshold for long-term depression (LTD)/LTP (Sheynikhovich et al., 2013). However, the exact mechanisms behind the impact of DA on memory functions are still largely unknown.

During formation of a novel memory, engaged neural networks can be widely distributed in the brain, including the hippocampus, cortex cerebri, amygdala, and additional regions. When the memory of an experience is of sufficient strength to be selected for long-term storage, timely signals from the global modulatory ascending systems are needed to inform these specific, but widely distributed, local systems that consolidation should take place. Here, we asked if communication between the local and global plasticity regulating systems is bilateral and, specifically, if the status of the key plasticity regulating Nogo signaling system may exert a local influence on dopaminergic function. Unexpectedly, local KCl-induced depolarization was found to lead to a dramatically reduced (80% less) KCl-induced increase of extracellular DA levels in MemoFlex mice as compared to control mice. This was the case despite normal density of TH-immunoreactive nerve terminals in MemoFlex mice as shown previously (Karlén et al., 2009), and confirmed in the present study (Figures 4A,B), normal levels of striatal DA (Karlén et al., 2009), and, as shown here, normal TH protein levels in the dorsal striatum, and no significant alteration of D1 or D2 receptor levels of the MemoFlex mouse striatum.

To determine if the strikingly impaired KCl-induced increase of extracellular DA was due to impaired release and/or increased reuptake, or increased extracellular metabolism, we also measured DAT levels and found no difference between control and MemoFlex striatum. Hence, it is unlikely that the decreased levels of depolarization-induced extracellular DA in MemoFlex mice are due to a more effective reuptake.

Amphetamine-induced release is dependent neither on neuronal activity nor on depolarization, but rather caused by leakage of DA from synaptic vesicles and leakage *via* DAT to the extracellular compartment (Heal et al., 2013). The fact that amphetamine-induced increase of extracellular DA was also significantly reduced in MemoFlex compared to control mice suggests that NgR1 overexpression may cause altered vesicle and/or DAT function.

To further address the cause of the observed effect of NgR1 overexpression on striatal DA release, we delivered exogenous DA to the location of the FAST recording electrode in the dorsal striatum. Our findings that T80 for exogenous DA was not affected by high NgR1 levels and that T100 was only modestly decreased, probably due to the overall higher concentration of DA in the control group, do not provide support for altered metabolism, reuptake, or metabolism of DA in the MemoFlex brain. Interestingly, the findings rather suggest that the DA nerve terminals are less sensitive to KCl depolarization and also less sensitive to amphetamine in the high NgR1 environment.

It has previously been shown that strong excitation (kainic acid) causes a temporary increase of the transcriptional activity of several genes involved in Nogo signaling, while the activity of one of them, NgR1, is first rapidly downregulated, soon to be followed by a sustained and marked upregulation to well above normal levels (Karlsson et al., 2017). Furthermore, the inability to downregulate NgR1 severely impairs the formation of lasting memories (Karlén et al., 2009). In MemoFlex mice, the NgR1 transgene is strongly overexpressed in forebrain areas, as driven by the CamKII promoter. In the MemoFlex striatum, the many fold increase of NgR1 can be ascribed to increased levels in both the corticostriatal projection neurons and the intrinsic striatal interneurons. Importantly, the DA neurons themselves express Nogo-A (Hunt et al., 2003; Kurowska et al., 2014; Schawkat et al., 2015). Thus, we hypothesize there could exist an increased Nogo–NgR1 interaction between DA nerve fibers and NgR1-expressing structures in the striatum.

Based on the current findings, there appears to be a synergistic interaction between the local and global plasticity systems. During the early stages of a plastic event, NgR1 is downregulated (Karlsson et al., 2017), thereby increasing the plasticity. At the same time, we hypothesize that the lower NgR1 levels could also cause an increase of DA release that further increases plasticity, such that the two systems “jump-start” the plastic event. Shortly afterward, NgR1 levels increase to above normal levels (Karlsson et al., 2017), which should stabilize the new memory, and at the same time drive down any additional DA release, securing stability of the new memory.

It has been shown that lack of Nogo-A results in a decrease of DA and 5-HT levels in the dorsal striatum by 62% and 75%, respectively (Willi et al., 2010), suggesting that the presence of normal Nogo-A levels is needed to maintain DA levels. Interestingly, if Nogo-A is only downregulated to about 50%, striatal DA levels are not affected (Tews et al., 2013), although motivational deficits are still observed (Enkel et al., 2014). It is possible that Nogo-A might be downregulated to compensate for the elevated NgR1 expression in forebrain neurons in MemoFlex mice, which in turn could decrease DA release. In contrast, and in line with our findings in a small sample of NgR1-overexpressing mice, DA release was significantly higher in NgR1 KO mice compared to that of controls.

Our findings using NgR1-overexpressing and NgR1 KO mice suggest that NgR1 levels in the striatum are negatively correlated to the amounts of DA that can be released by neuronal depolarization. Thus, we hypothesize that activity-driven early decrease of NgR1 would increase plasticity not only by lowering the sensitivity to local Nogo-A but also by allowing larger amounts of plasticity-enhancing DA to be released. Taken together, the data suggest that local and global plasticity regulating systems may operate in synergy to create temporary plasticity windows in specific regions during defined time periods.

## Data Availability Statement

The raw data supporting the conclusions of this article will be made available by the authors, without undue reservation.

## Ethics Statement

The animal studies were reviewed and approved by the Stockholm Animal Testing Ethics Committee.

## Author Contributions

EA, TK, and LO designed the research and wrote the article. EA, SG, and AB performed experiments. EA, SG, AB, and TK analyzed the data. All authors contributed to the article and approved the submitted version.

## Conflict of Interest

The authors declare that the research was conducted in the absence of any commercial or financial relationships that could be construed as a potential conflict of interest.

## Supplementary Material

SUPPLEMENTARY FIGURE 1 Potassium chloride (KCl)-induced dopamine (DA) release and reuptake parameters in Nogo receptor 1 (NgR1) KO and littermate controls. **(A)** Mean values of DA released by five KCl ejections. **p* < 0.05 as detected by two-tailed Student’s *t*-test, expressed as mean ± SEM. Controls: black; NgR1 KO: blue. NgR1 KO: *n* = 4 males; controls: *n* = 4 males. **(B)** Raw traces of DA release after KCl in NgR1 knock-out and control mice.
